# More than chest pain: A case of oesophageal foreign body ingestion

**DOI:** 10.4102/safp.v66i1.5942

**Published:** 2024-08-14

**Authors:** Mohammad M.A. Agbabiaka, Idris T. Akinwande, Chika K. Egenasi, Mathew O. Benedict

**Affiliations:** 1 Primary Health Care Corporation, Doha, Qatar; 2 Health and Medical Sciences Office, College of Medicine, Qatar University, Doha, Qatar; 3 Department of Family Medicine, Faculty of Health Sciences, University of the Free State, Bloemfontein, South Africa

**Keywords:** fishbone ingestion, chest pain, ECG, CT, cardiac, oesophageal, foreign body, mediastinum

## Abstract

**Background:**

Physicians often focus on possible cardiac causes in patients presenting with chest pain. However, this case highlights a patient who presented with chest pain caused by ingestion of a foreign body after an uneventful meal eaten an hour prior to presentation. The fishbone was discovered after imaging. The article aims to raise awareness regarding the potential origins of chest pain, highlighting that it may stem from non-cardiac conditions.

**Methods:**

The methodology employed in this study involved conducting a case study that meticulously examined the repercussions and management strategies associated with foreign body ingestion.

**Results:**

The case report delineates the scenario of a 27-year-old male patient who inadvertently ingested a fishbone during a routine meal. It details the swift decline in clinical status, the meticulous diagnostic procedures employed, the subsequent management strategies implemented and the ultimate discharge of the patient in a stable condition.

**Conclusion:**

This case highlights the importance of comprehensive history taking and considering a wide range of causes of chest pain when evaluating a patient. The foreign body ingested with the resulting cardiac complications could have been fatal if not promptly diagnosed.

**Contribution:**

This study contributed to advancing awareness surrounding foreign body ingestion, shedding light on potential complications and offering valuable insights into effective management strategies.

## Introduction

Chest pain is a common complaint; it includes various differential diagnoses and constitutes a frequent reason for visits to the emergency department.^1^ When assessing chest pain, the healthcare provider must consistently explore other potential life-threatening causes of discomfort.^1^ Common causes of chest pain can be cardiac or non-cardiac, and chief among them are acute coronary syndromes, Gastro-esophageal reflux, pulmonary embolism, pericarditis, tension pneumothorax, ruptured peptic ulcer, musculoskeletal and pleuritic chest pain.^1,2^ Cases of acute chest pain caused by oesophageal foreign bodies are relatively rarely reported.^3^

Foreign body and food impaction rank among the prevalent gastrointestinal complaints observed in emergency departments. Accidental ingestion of fish bones is a frequently encountered issue in emergency departments, particularly in regions like Asia and the Mediterranean, where the consumption of unboned fish is common.^4,5^ The estimated annual incidence of food impaction is 13.0 per 100 000 and can be seen in children and adults.^6^ Approximately 80% of patients presenting with an oesophageal foreign body in emergency departments are children.^6,7^ These incidents typically involve unintentional ingestion of small objects like coins, pins, needles, batteries, toy components, crayons, fish and chicken bones, large food boluses and jewellery.^6^ Among these, coins are the most frequently ingested foreign bodies by children. While most children exhibit normal anatomy, those with abnormalities such as eosinophilic esophagitis, prior oesophageal atresia repair or prior Nissen fundoplication are at an increased risk of impactions.

In adults, it tends to occur in older adults above the age of 70 years.^7^ They are mostly accidental in 95% of cases, with 80% to 90% of cases affecting the distal oesophagus and being associated with anatomical or motor irregularities such as diverticula, webs, rings, strictures, tumours, eosinophilic esophagitis, achalasia, scleroderma or oesophageal spasms.^6^ Additional risk factors encompass self-harm, alcohol or substance abuse, lack of teeth and the use of dentures.^5^ Obstruction most frequently occurs at the thoracic inlet, at the level of the clavicles and the cricopharyngeus muscle, at the level of the carina and aortic arch and the level of the oesophagogastric junction.^5^

It is estimated that 1500 people die annually from upper gastrointestinal foreign body ingestion because of complications such as perforations, obstructions and soft tissue infections.^7^ The anatomical proximity of the gastrointestinal tract, particularly the distal oesophagus, to adjacent structures can result in damage to surrounding tissues if a foreign body perforates. This may give rise to complications such as pneumo-mediastinum, pericarditis/tamponade, pneumothorax or development of trachea-oesophageal or aorta–oesophageal fistulas, among other potential issues. The symptoms often mimic other common ailments and pose a diagnostic challenge. It is, therefore, important to have a high index of suspicion in patients with relevant signs and symptoms to aid prompt investigations and diagnosis, thereby reducing the risk of complications. We present a case of cardiac tamponade caused by oesophageal perforation of a fish bone and associated diagnostic challenges to highlight this rare occurrence.

## Case study

A 27-year-old man presented at the local primary health centre with an hour’s history of left-sided chest pain that radiated to the neck. He had noticeable difficulty breathing and was sweating. There was no history of nausea and vomiting. He ate a meal of rice and fish prior to the presentation. He was a smoker, smoking about 20 cigarettes per day. There was no family history of heart disease. He had tachycardia and tachypnoea on examination, with a pulse rate of 162 beats per minute (bpm) and a respiratory rate of 24 cycles per minute. His initial blood pressure (BP) was 132/88 mmHg, oxygen saturation was 100% on room air and the temperature was 37 °C; his heart sounds and the rest of his chest examination were normal.

**FIGURE 1:**
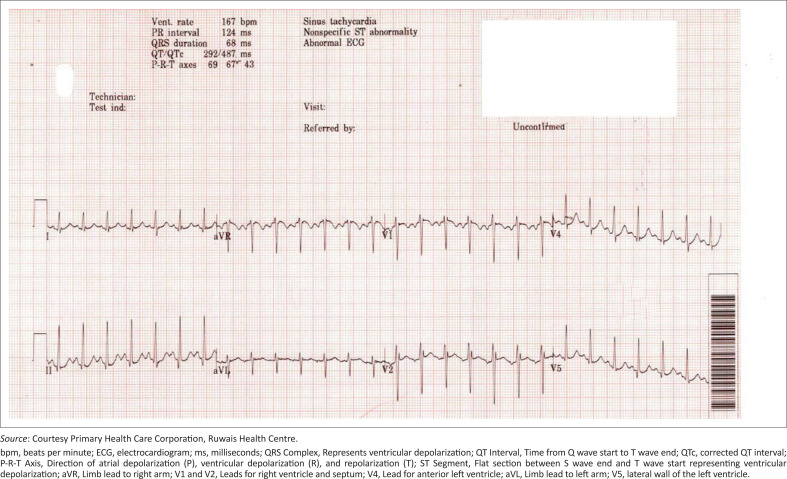
Electrocardiogram showing sinus tachycardia and electrical alternans.

Electrocardiogram (ECG) on presentation showed sinus tachycardia (167/min) and electrical alternans with no T wave or ST changes (Figure 1). Intravenous paracetamol, normal saline infusion and nebulised saline were administered at the primary care centre to alleviate his pain and for symptom control. He was transferred as an emergent case to a secondary care facility.

**FIGURE 2:**
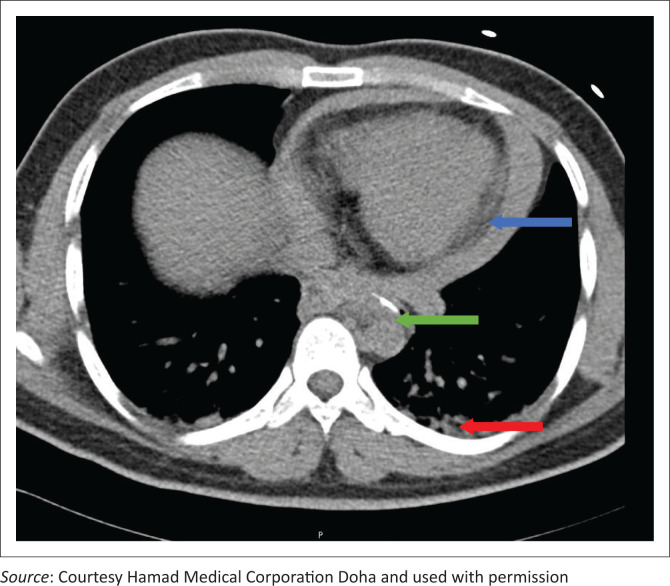
Computed tomography scan showing pericardial effusion (blue arrow), hook-shaped radio-opaque shadow (green arrow) and blood in the thorax (red arrow).

Upon arrival at the secondary care facility, he exhibited hypotension, with his BP dropping to 80/50 mmHg. Subsequent examinations included an unremarkable chest X-ray, and a bedside echogram revealed mild pericardial effusion. A computed tomography (CT) angiogram of the aorta was then conducted, uncovering a curvilinear radio-opaque shadow with a hook-shaped appearance measuring 1.1 cm in the thoracic oesophagus. The imaging also indicated an associated pericardial effusion, with no evidence of pneumothorax or aortic dissection (Figure 2).

He had an emergency oesophagus-gastro-duodenoscopy, which showed erosion in the oesophagus at 35 cm from the incisors, but no evidence of a foreign body was seen. The team proceeded to a thoracoscopy, which showed a mediastinal haematoma, very close to the descending aorta, with blood in the thorax. A chest drain was then inserted. Differential diagnoses at this point were ischaemic heart disease, aortic dissection, foreign body perforation and haemothorax.

The vascular surgeons operated on him the following day, and he was found to have a left posterior atrial wall injury in addition to the earlier noted oesophageal injury. A foreign body was removed from the oesophageal wall and confirmed to be a fish bone. The mediastinal haematoma was drained, and the blood in the thorax was evacuated. The pericardium was repaired by suturing. The patient spent a total of 13 days in the hospital, including his intensive trauma unit (ITU) stay. He made a complete recovery and was discharged in a stable condition.

## Discussion

Compared to middle-aged or older patients, chest pain in a younger adult poses a diagnostic challenge because of the varied differential diagnoses. An article by Johnson and Ghassemzadeh on chest pain stated that it is necessary to rule out potentially fatal pathological causes of chest pain before considering benign causes.^1^ Stepinska et al. stated that chest pain can be caused by a variety of disorders, both cardiac and non-cardiac, ranging from life-threatening syndromes such as acute coronary syndrome to harmless conditions.^2^ In the index case, the chest pain experienced was cardiac but was of gastrointestinal origin (perforation through the oesophagus). The history of recent ingestion of fish perhaps heightened the possibility of fishbone ingestion. If not for the availability of a bedside echogram, which revealed cardiac pathology, that is, pericardial effusion, the focus would have been limited to gastrointestinal aetiology. This emphasises the importance of point-of-care ultrasonography (POCUS) in acute presentations, especially where formal imaging may delay diagnosis.^8^

Oesophageal foreign body ingestion is a common presentation in the emergency department.^4,5^ The majority of foreign body ingestions are unwitnessed and resolved without requiring the involvement of a healthcare professional,^5^ with only about 10% – 20% requiring endoscopic removal and hardly 1% needing surgery.^4^ Some patients can be asymptomatic; others may present with symptoms such as epigastric pain, vomiting, dysphagia, pharyngeal discomfort and chest pain.^4^ Early in the management, it is important to transfer these patients to healthcare facilities with adequate diagnostic and management capabilities.

This case involved a young adult without any prior history of oesophageal pathology. The clinical history and presentation suggested a foreign body ingestion. X-rays were accessible and appeared to be the next logical course of action, revealing relatively normal findings. About 83% of ingested foreign bodies exhibit radiopacity.^5^ Mathew et al., in a study on clinical presentation, diagnosis and management of aerodigestive tract foreign bodies, reported that traditional neck radiography (lateral view), because of its cost-effectiveness and widespread accessibility, is frequently the initial imaging method employed in assessing individuals who have inadvertently swallowed a fishbone.^4^ Nevertheless, the diagnostic effectiveness of X-rays in detecting fish bones is uncertain and controversial, given a reported false-negative rate of 47% and an exceptionally low sensitivity of 25.3%. Because of the risk of aspiration, an oral contrast examination should not be performed,^4^ which was perhaps the reason for its omission in this case. For radiolucent foreign entities in situations where there is a high index of suspicion, a CT scan may be performed.^9^ A CT scan has a high sensitivity for foreign bodies and assesses for complications such as perforation mediastinitis, arterial injury, lung damage and abscess formation.^4^ This supports the CT angiogram findings in our case, which identified the presence of a hook-shaped radio-opaque shadow in the oesophagus and the associated complications. Most cases of oesophageal food impaction are treated by flexible endoscopy (suitable for pointed objects), which should not be delayed by more than 24 h after presentation because of the risk of complications and has been reported to have a success rate of 88.5% – 100%.^9^ In a case where the fishbone cannot be located by endoscopy, an endoscopic mucosal incision may be required.^10^ Surgery becomes the ultimate recourse in cases of endoscopic failure or severe complications.^10,11^

In our case, the endoscopic procedure could not locate the suspected fishbone, which may be because it was deeply embedded in the oesophageal wall, thus the need for surgical intervention.

The goals of surgery in the index case were to control the bleeding, drain the thorax, repair the pericardium, drain the mediastinal haematoma and repair the oesophageal defect.

In conclusion, foreign body ingestions such as fishbones are common presentations in an emergency department. However, perforation with sequel cardiac complications is a rare occurrence.^12^ A delay or missed diagnosis can lead to increased morbidity and mortality for the patient. The patient must be correctly diagnosed and referred to a facility with the necessary resources, trained personnel and a multidisciplinary team to manage such complicated medical conditions.

As most cases present initially at the primary care facilities,^13,14^ the primary care physicians must be skilled in the use of POCUS as it aids in prompt confirmation or exclusion of potentially fatal diagnoses.^15,16^
